# Supplementary material for: Genetic and metabolic regulation of Mycobacterium tuberculosis acid growth arrest
Source: Sci Rep. 2018 Mar 8;8:4168. doi: 10.1038/s41598-018-22343-4 (PMC5843633; doi:10.1038/s41598-018-22343-4)
Supplement: Supplementary file 1 — Supplementary Figures [file 41598_2018_22343_MOESM1_ESM.pdf]

Supplemental Figures

Genetic and metabolic regulation of *Mycobacterium tuberculosis* acid growth arrest

Jacob J. Baker[1] and Robert B. Abramovitch[1]*

[1]Department of Microbiology and Molecular Genetics, Michigan State University, East Lansing, Michigan, 48824, United States.

Supplemental Figures

Supplemental Figure 1. Wild type growth phenotypes are restored in the Δ*pckA* mutant with addition of glycerol. Growth of WT, Δ*pckA* mutant, and complemented strain (Δ*pckA*-Comp) was measured over time in minimal media supplemented with the indicated carbon sources. A-D) Δ*pckA* maintains growth arrest on glycerol at pH 5.7, but is deficient for growth on pyruvate, acetate, and succinate as indicated by a decrease in OD_{600} over time. E-G) Addition of glycerol as a second carbon source restores WT levels of growth in the Δ*pckA* mutant on acetate, pyruvate, and succinate.

Supplemental Figure 2. Growth defects of Δ*icl1/2* mutant are not restored with the addition of glycerol as a second carbon source. Growth of WT and Δ*icl1/2* mutant was measured over time in minimal media supplemented with the indicated carbon sources. A-B) Δ*icl1/2* achieves mild bacterial growth on both glycerol at pH 5.7 and pyruvate at pH 7.0 and pH 5.7. This low-level bacterial growth is increased compared to WT at pH 5.7 on glycerol and reduced compared to WT on pyruvate. C) The Δ*icl1/2* mutant is unable to grow with acetate as single carbon source. D-E) Addition of glycerol as a second carbon source does not restore growth of the Δ*icl1/2* mutant.

Supplemental Figure 3. Growth of Δ*icl1/2* mutant at acidic pH is not affected by addition of vitamin B12. Summary data showing the OD_{600} measured on day 12 of growth curves performed in minimal media containing either glycerol or pyruvate as single carbon sources, buffered to pH 7.0 or pH 5.7, and with or without supplementation of vitamin B12. No difference in Mtb growth was observed with supplementation of vitamin B12.

Supplemental Figure 4. Metabolic profiling of Mtb Erdman wildtype and Δ*icl1/2* mutant strains on minimal media agar plates buffered to pH 7.0 or pH 5.7 and containing either

glycerol or pyruvate as a single carbon source. Metabolite concentration is reported as the relative peak area per µg of protein for each treatment. Error bars represent the standard deviation. Statistical analyses are included in Supplemental Table 1.

Supplemental Figure 5. Metabolic profiling of Mtb Erdman wildtype and ∆*pckA* mutant strains on minimal media agar plates buffered to pH 7.0 or pH 5.7 and containing either glycerol or glycerol and pyruvate. Metabolite concentration is reported as the relative peak area per µg of protein for each treatment. Error bars represent the standard deviation. Statistical analyses are included in Supplemental Table 1.

Supplemental Figure 6. Nitrate decreases Mtb growth on pyruvate specifically at acidic pH but has little effect on succinate secretion. A. Growth of Mtb in minimal medium with pyruvate as a single carbon source buffered to pH 7.0 or pH 5.7 with or without 5 mM sodium nitrate ($NaNO_3$). Mtb growth at pH 7.0 is not affected by addition of nitrate, but at pH 5.7 addition of nitrate causes a ~50% inhibition of growth. **B.** Secretion of succinate is decreased ~40% with the addition of nitrate at pH 5.7 with pyruvate as a single carbon source, but this could be due to the decreased growth in this condition.

Supplemental Figure 7. Mtb utilizes glycerol for anabolic metabolism during acid growth arrest.

TLC images showing relative abundance of TDM and TMM (**A**), TAG (**B**), and SL (**C**) at pH 7.0 and pH 5.7. Barely detectable quantities of ^{14}C-glycerol were incorporated into TAG.

Supplemental Figure 8. Genetic screen to identify mutants with enhanced growth at acidic pH. Mutants able to form colonies on agar plates buffered to pH 5.7 containing glycerol as a single carbon source were isolated and confirmed as enhanced acid growth (*eag*) mutants

by measuring growth in liquid culture conditions of acid growth arrest. **A.** Compiled growth phenotypes for all isolated mutants. Each dot represents an individual mutant, with the fold change in OD_{600} from day 0 to day 9 reported. The dotted line represents the fold change observed in the WT control. **B.** Failed phenotypic complementation of a transposon *eag4* mutant, Tn:MT3159. The mutant was complemented by introduction of an integrative plasmid containing an intact version of the disrupted gene, MT3159, and its native promoter. WT, wild type Mtb. **C.** Genetic complementation of Tn:MT3159. Quantitative real time PCR revealed that the complementation strain (Comp) of Tn:MT3159 can restore the decreased expression of MT3149 transcript levels in the Tn mutant.

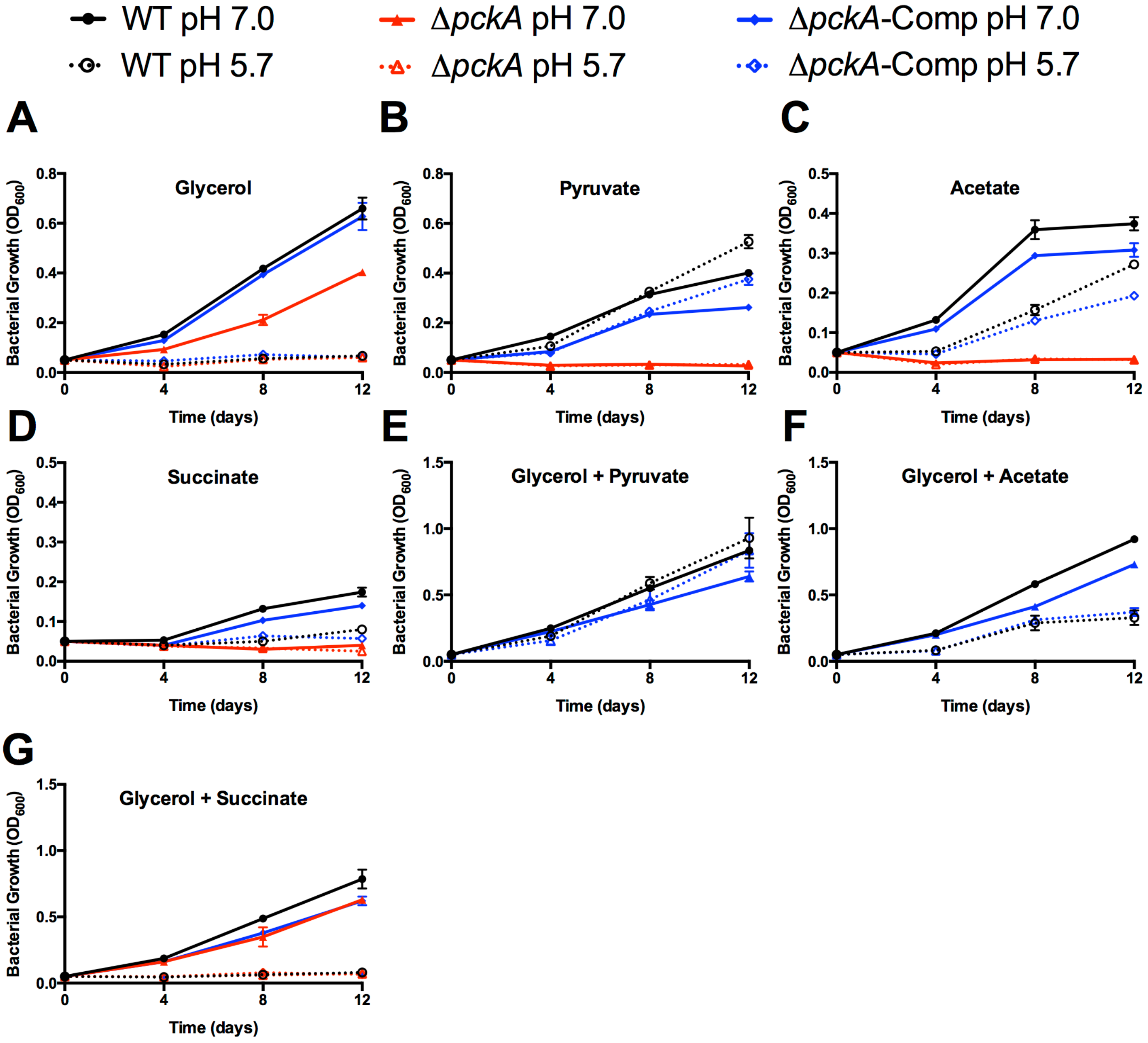

Supplemental Figure 1

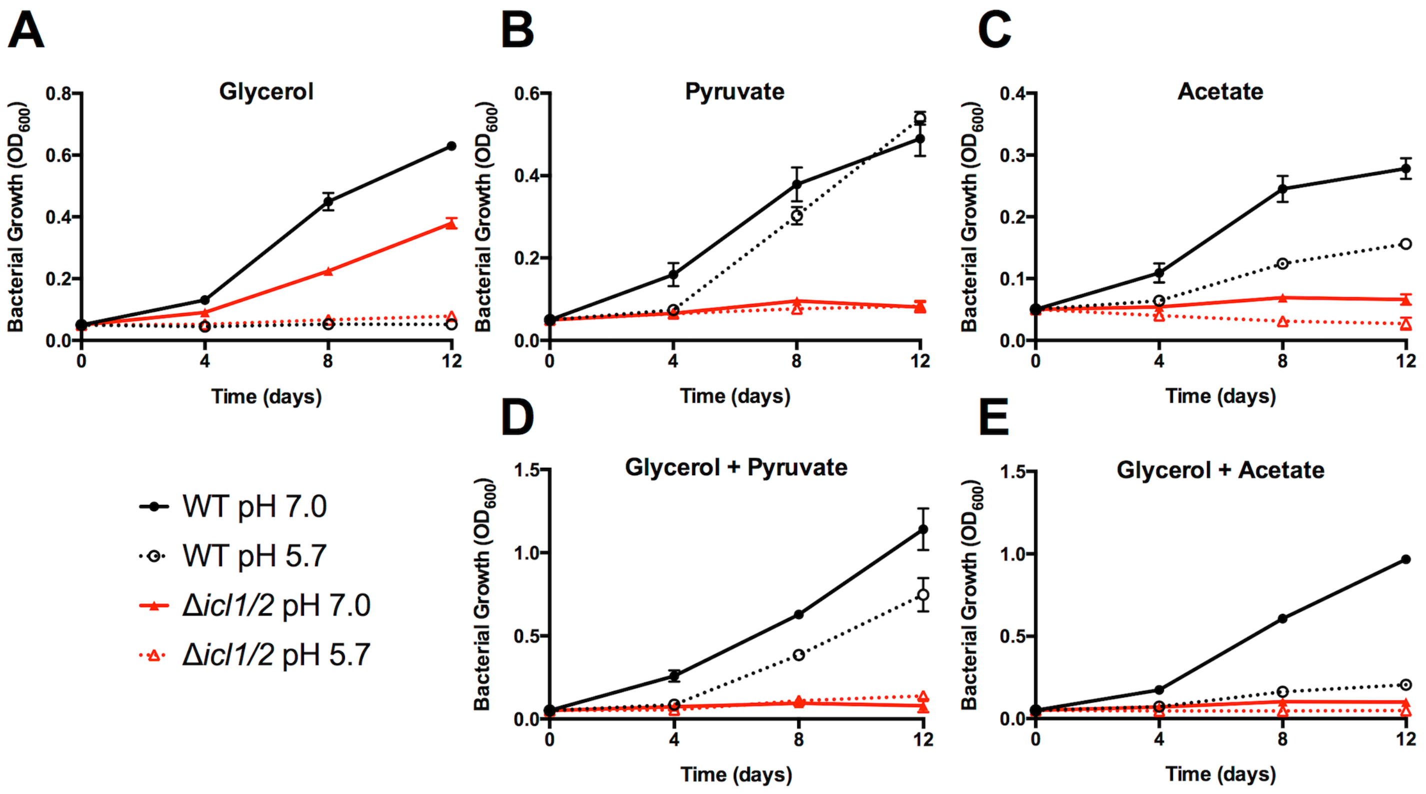

Supplemental Figure 2

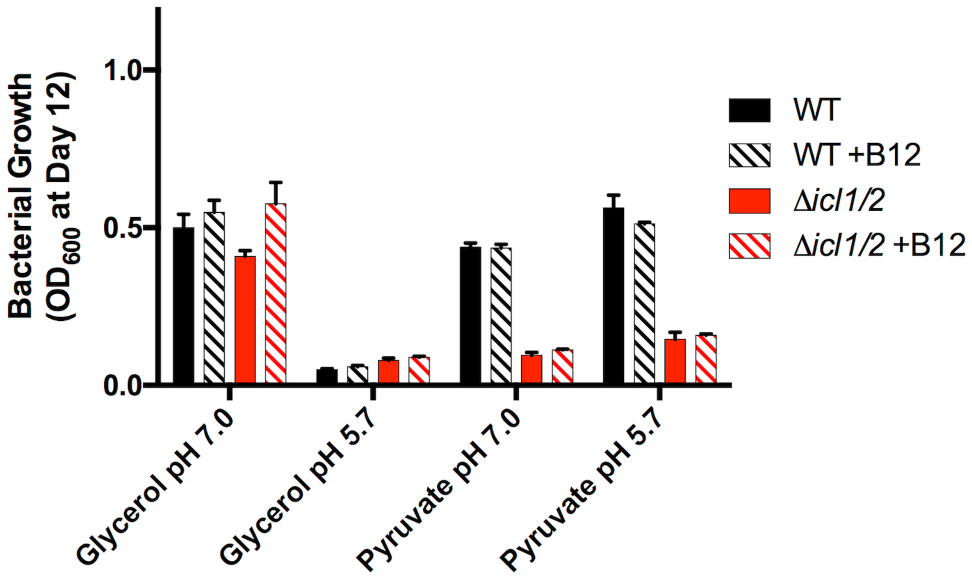

Supplemental Figure 3

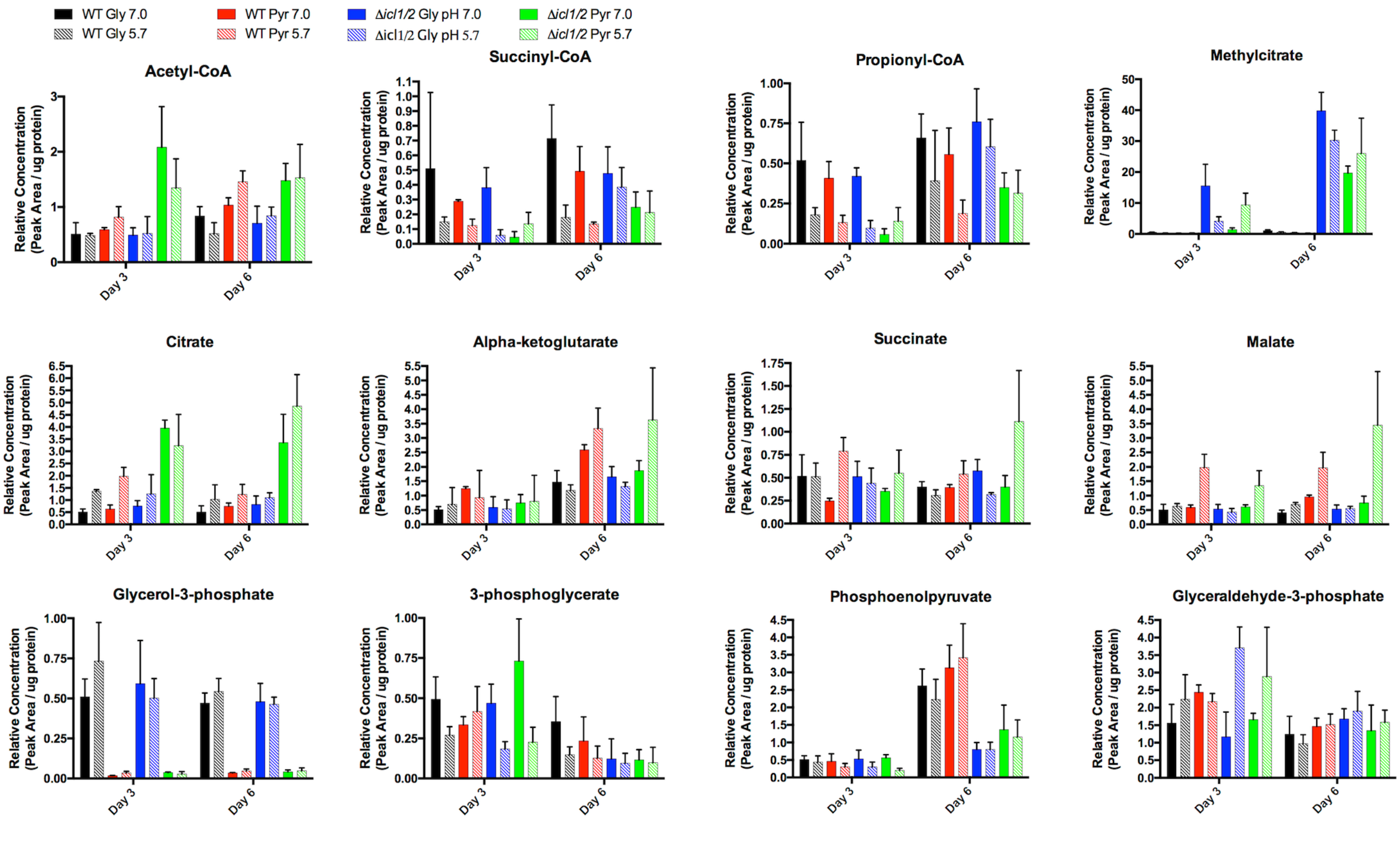

Supplemental Figure 4

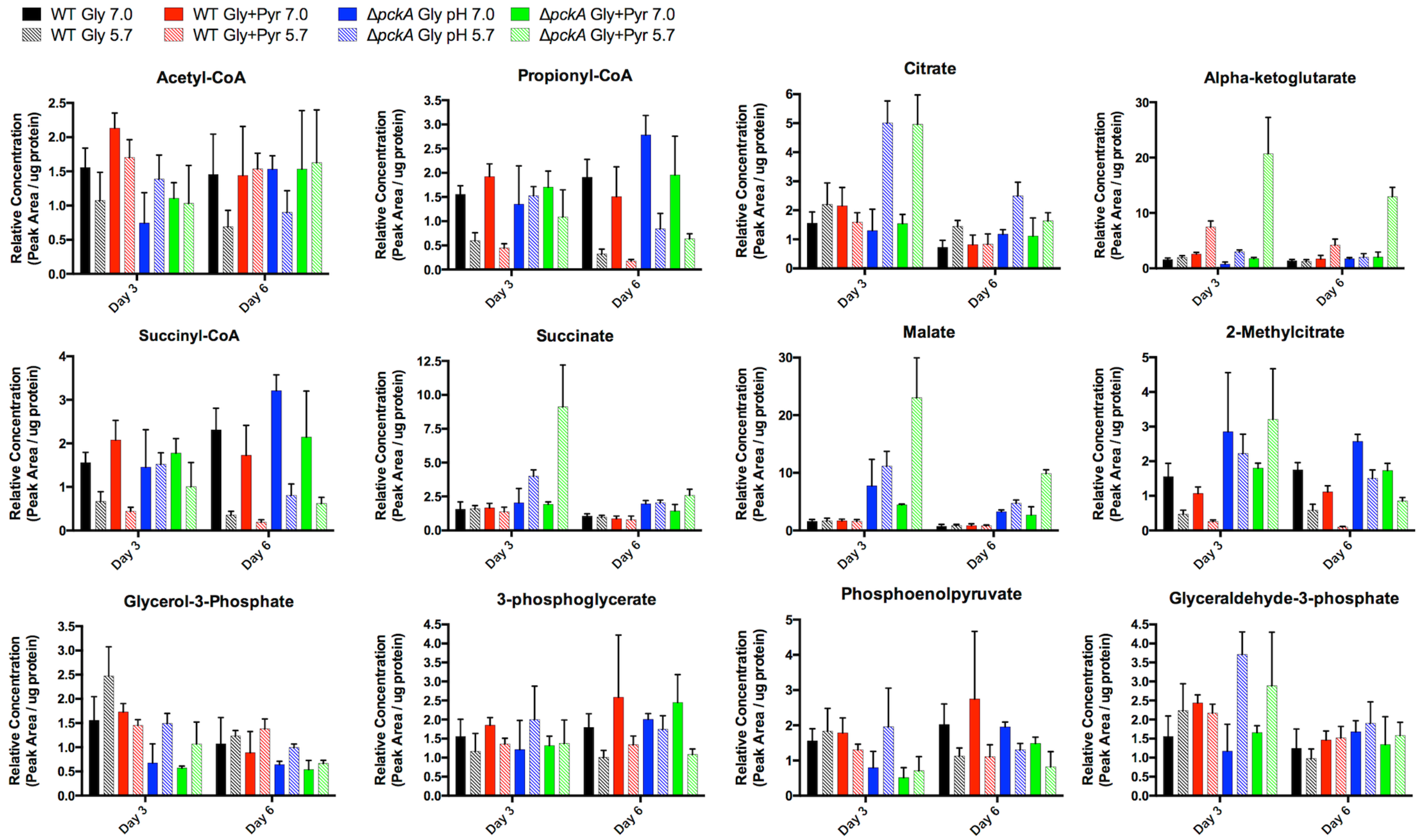

Supplemental Figure 5

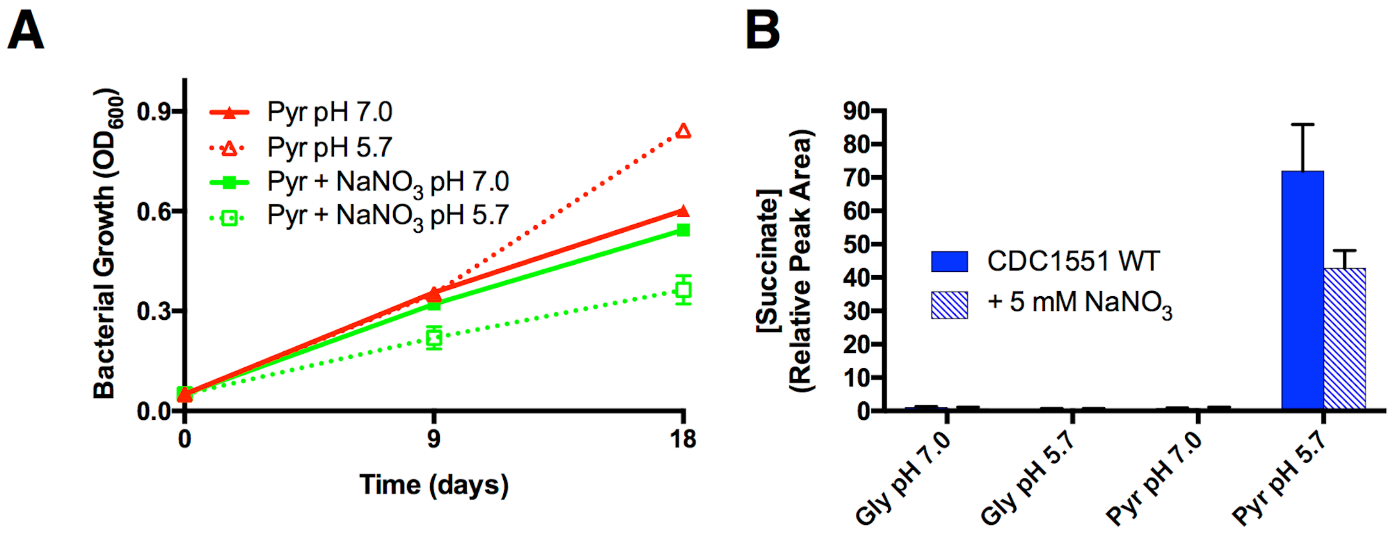

Supplemental Figure 6

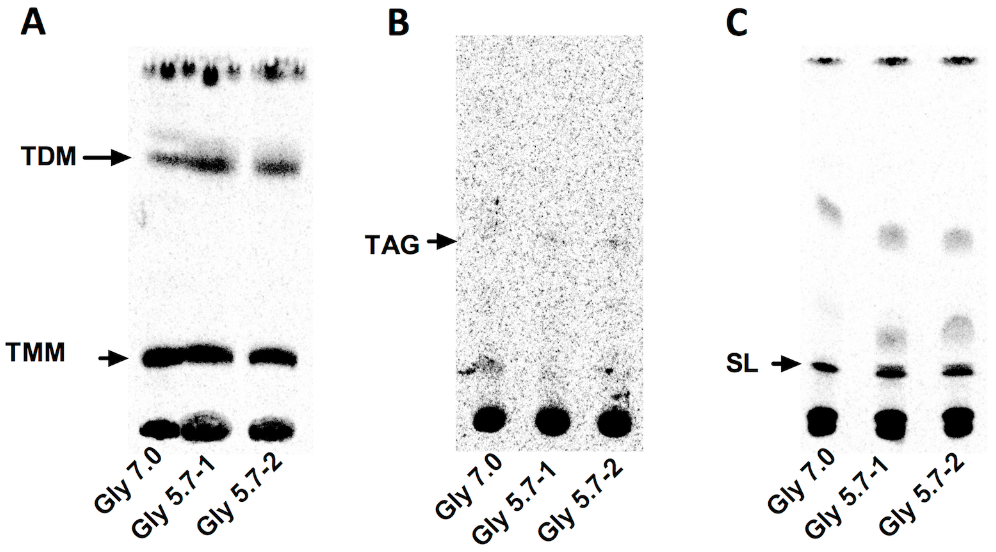

Supplemental Figure 7

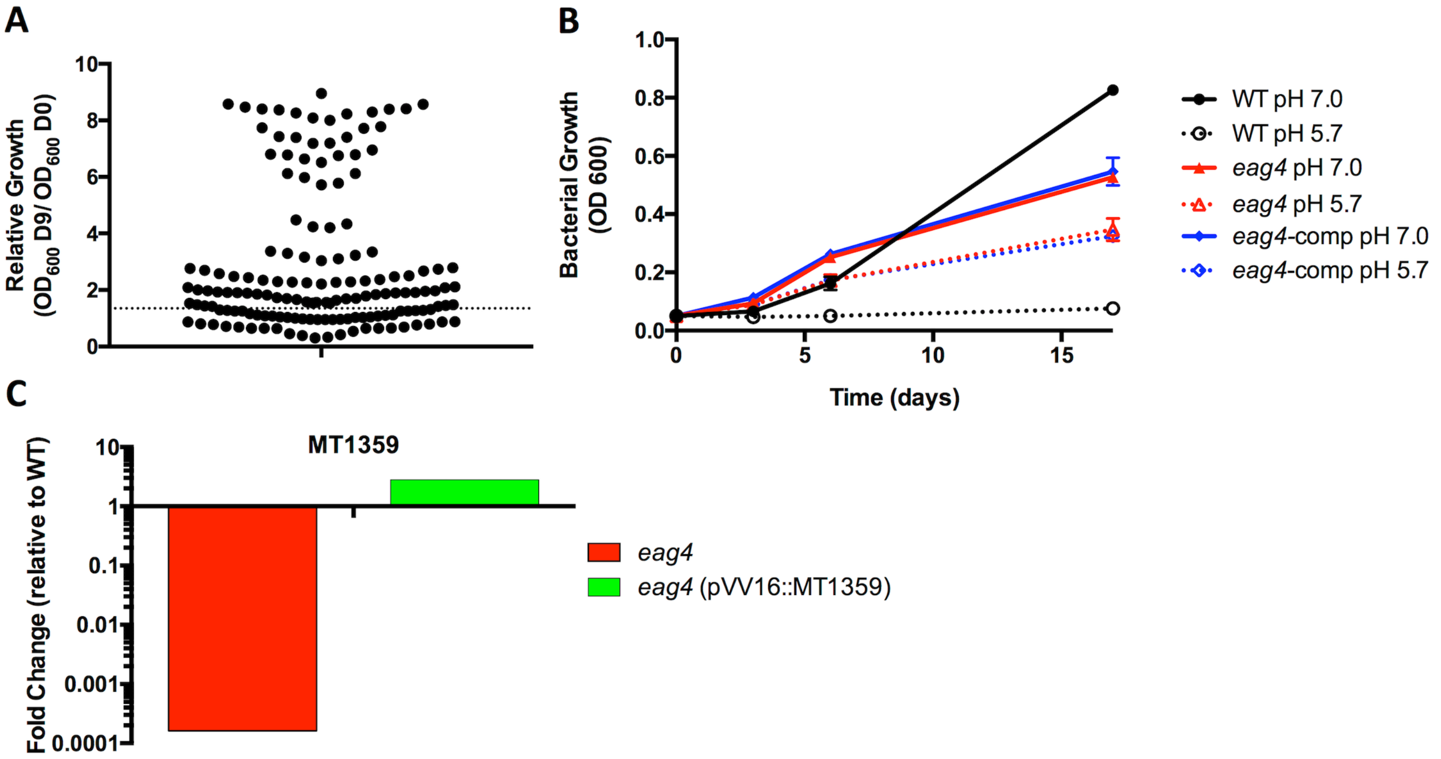

Supplemental Figure 8
